# Distal amputations for the diabetic foot

**DOI:** 10.3402/dfa.v4i0.21288

**Published:** 2013-07-16

**Authors:** Aziz Nather, Keng Lin Wong

**Affiliations:** Department of Orthopaedic Surgery, University Orthopaedic, Hand and Reconstructive Microsurgery Cluster, National University Health System, Singapore

**Keywords:** diabetic foot infections, amputations, diabetic neuropathy

## Abstract

Minor amputations in diabetic patients with foot complications have been well studied in the literature but controversy still remains as to what constitutes successful or non-successful limb salvage. In addition, there is a lack of consensus on the definition of a minor or distal amputation and a major or proximal amputation for the diabetic population. In this article, the authors review the existing literature to evaluate the efficacy of minor amputations in this selected group of patients in terms of diabetic limb salvage and also propose several definitions regarding diabetic foot amputations.

One of the most valuable strategies for managing the diabetic foot is to prevent the development of foot complications since neuropathic foot ulceration can often lead to loss of a limb due to a major amputation, i.e. below the knee amputation (BKA). For this to be achieved, patients diagnosed with diabetes mellitus must be subjected to early annual foot screening programs (1). Once a diabetic foot complication has developed, the next best strategy is to treat this complication early in a hospital setting by a multidisciplinary diabetic foot team (2, 3). The objective of early and efficacious treatment is to achieve limb salvage in order to avoid the loss of a limb from a major amputation.

However, controversy still remains in the existing literature as to what constitutes successful or non-successful diabetic limb salvage as well as a definition of a minor or distal amputation and a major or proximal amputation. Izumi et al. (4) referred to a Syme amputation as major amputation and in a study by Evans et al. (5), no Syme amputation was included where the authors compared forefoot and midfoot amputations to BKA. Svensson et al. (6) compared a BKA group with an above-the-ankle amputation.

Based on the current literature, the authors propose the following definitions regarding the terms of distal or minor amputation versus a proximal or major amputation (Table 1). In addition, the terms successful versus non-successful amputations for diabetic limb salvage are still needed to be defined based on the amputation level, functionality, recurrence of ulceration and/or amputation and in larger cohort studies. The ASEAN Plus Expert Group Forum for the Management of Diabetic Foot Wounds that was held in Singapore on November 10, 2002, developed clinical practice guidelines for the management of diabetic foot wounds and has adopted these criteria. The senior author of this article is the chairman of this forum and the workgroup includes two experts each from Indonesia, Malaysia, Philippines, Singapore, Sri Lanka, and Thailand.

**Table 1:** Classification of various diabetic foot surgeries into major or minor amputations.

Amputation level	Distal or minor amputation (tibial weight-bearing stump is preserved)	Proximal or major amputation (tibial weight-bearing stump cannot be preserved)
Forefoot	Toe disarticulation	
	Ray (metatarsal and toe)	
	Transmetatarsal	
Midfoot	Lisfranc	
	Chopart	
Hindfoot	Syme	
	Boyd	
	Pirogoff	
	Modified Pirogoff	
Trans-tibial		Below the knee
Through the knee		Gritti stokes
Trans-femoral		Above the knee
Hip		Hip disarticulation

The first amputation for patients with diabetic foot complications should preferably be a minor (distal) amputation. When a major (proximal) amputation, such as BKA is performed, the mortality rate is significantly higher than when a minor amputation (such as ray) is performed (4, 5). Izumi et al. (4) reported a significant difference in mortality with the hazard rate being 1.6 times in major amputees compared to ray amputees. Evans et al. (5) found that 80% of minor amputees were still alive after 2 years; 73% of minor amputees preserved their lower limb; and 64% were still fully ambulatory. In the BKA group, 52% died within 2 years and only 64% of patients ambulated with a prosthetic limb (5). Svensson et al. (6), in a study of 410 patients undergoing minor amputations, found that limb salvage could be achieved in almost two-thirds of patients.

## Distal or minor amputations for the diabetic foot

### Forefoot amputation

Toe disarticulations can be quite challenging for the surgeon when dealing with the remaining cartilage of the involved metatarsal head and possible complications of residual osteomyelitis. Atway et al. (7) reported a positive margin culture in three out of 13 patients (23.1%) who had a toe disarticulation.

Ray amputation, which involves the excision of the toe and part of the metatarsal, provides a more viable option of ensuring an adequate surgical debridement of the septic margins. Indications may include a wet or dry gangrene of a toe, osteomyelitis of the metatarsal head and/or proximal phalanx, septic arthritis of the metatarsophalangeal joint (MTPJ) and gross infection of the toe. Suggested inclusion criteria for this type of amputation may include one or two palpable pedal pulses, ankle brachial index (ABI) ≥ 0.8 and toe brachial index ≥ 0.7.

Borkosky et al. (8) reported a 19.8% incidence of re-amputation in patients with diabetes and peripheral sensory neuropathy undergoing partial first ray resection. Wong et al. (9) reported a 70% success rate of ray amputation in a cohort of 150 patients with diabetic foot problems. Absence of pulses, delayed capillary filling, high erythrocyte sedimentation rate, high creatinine and high neutrophil counts were found to be predictive factors for a poor clinical outcome (9).

Indications for transmetatarsal (TMA) amputation may include wet or dry gangrene involving only the forefoot and/or infection involving the forefoot while the inclusion criteria are the same as those mentioned above required for a ray amputation. Brown et al. (10) in a retrospective study of 21 patients reported a high functioning level and durability of the stump in patients undergoing TMA and concluded that it provides an ambulatory advantage. However, TMA has been reported to give significant complication and failure rates. Anthony et al. (11) reported 82% of patients requiring further surgery while Pollard et al. (12) reported a wound-healing rate of only 54%.

Amputation at the metatarsal level causes a muscular imbalance due to resultant equinovarus deformity from unopposed action of gastrocnemius, tibialis anterior, and tibialis posterior tendons, which is coupled with the deficiency of the muscular tension of the extensor tendons (12). Adjunctive soft tissue procedures such as tendo-Achilles lengthening and split tibialis anterior tendon transfer for muscular imbalance are needed to correct for the equinovarus deformity. In addition, special footwear modifications are needed to reduce complication rates (13).

### Midfoot amputation

**Fig. 1:**
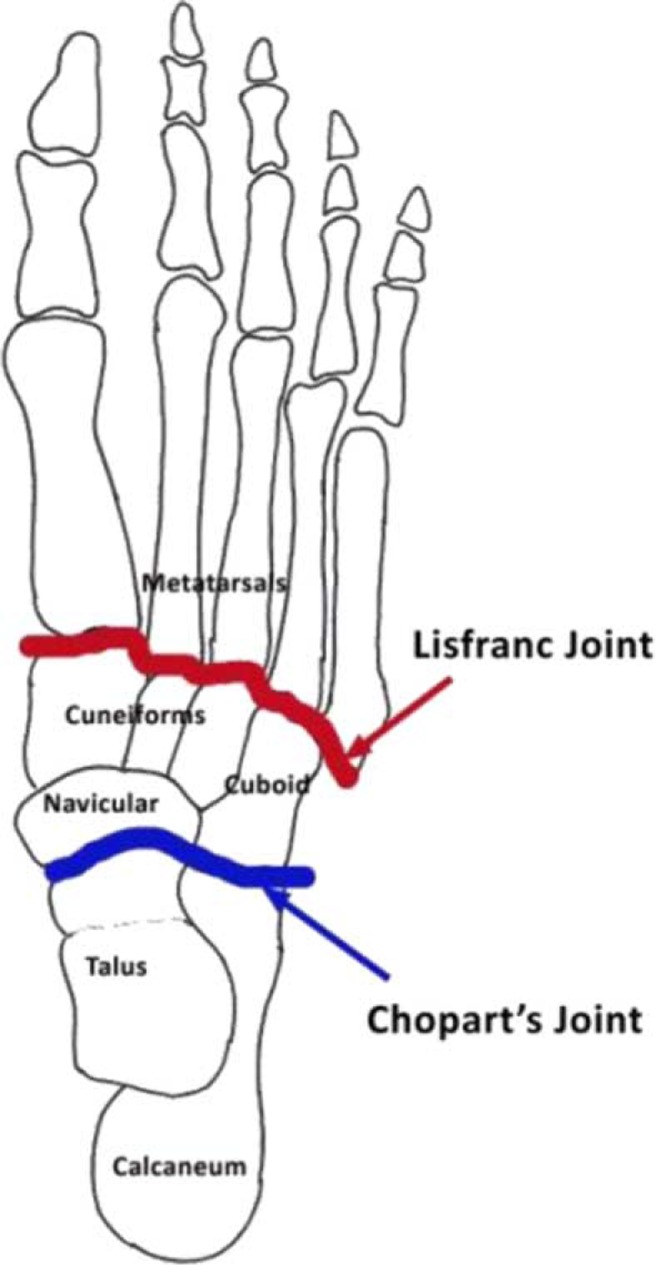
Diagram demonstrating the Lisfranc's and Chopart's joints.

Lisfranc's disarticulation is a disarticulation through the tarsometatarsal joint, while Chopart's disarticulation is a disarticulation through the talonavicular and calcaneocuboid joints leaving only the hindfoot (talus and calcaneum) behind (Fig. 1). These amputations are rarely performed in diabetic foot infections due to high failure rate and the proximity of infected tissue to the heel pad. However, Brown et al. (10) reported high ambulatory levels for Chopart's disarticulation in his series of 10 patients. This suggests a favorable advantage for patients to ambulate if peri-operative and post-operative complications could be avoided.

Elsharawy (14) studied the outcome of midfoot amputations in diabetic gangrene in his cohort study of 32 patients. There were wound-healing complications in eight patients (27%), which necessitated a BKA. Successful limb salvage, which was defined as a stump with functional ambulation, was seen in only 30 patients (67%) (14). A systematic review of the existing literature was conducted by Schade et al. to identify any factors that may be associated with a successful Chopart amputation in diabetic foot problems (15). The efficacy of tendinous and/or osseous balancing could not be assessed due to lack of comparable techniques, highlighting the paucity of literature in this field (15).

### Hindfoot amputation

This category included the amputations as shown in Table 1 and has the indications and inclusion criteria as mentioned in the forefoot amputation category. Syme's amputation has been advocated for trauma cases (16); however, with strict selection criteria, Syme's amputation can give good results in patients with diabetic foot infections (17). It is well known that Syme's amputation should be reserved for patients with at least a palpable posterior tibial pulse and an ankle-brachial index of more than 0.5 (17–19). There are several disadvantages to performing a Syme's amputation. This includes instability of the calcaneal flap due to poor adherence of the soft tissue of the calcaneal flap to the tibial surface. Also, the dissection of the calcaneum from the underlying flap in a Syme's amputation may lead to devascularization of the flap (20). A third disadvantage is that the Syme's amputation with excision of the calcaneum leads to a shorter stump. This causes significant limb length discrepancy, which makes walking barefoot difficult (21).

The Boyd and Pirogoff amputation is designed to give better results than the Syme's amputation (21–24). In the Boyd and Pirogoff amputation, the tibio-calcaneal bony fusion gives added stability to the flap. There is also reduced devascularization of the flap since the calcaneum is not dissected. Limb length discrepancy is also minimized. Along with a stable full weight-bearing stump due to the tibio-calcaneal fusion, the additional length makes it easier for the patient to walk without a prosthesis (23). In addition, a part of the medial and lateral malleolus preserved in these amputations makes it easier for prosthesis to be fitted. The prosthesis can be worn with less friction and is more rotationally stable compared to a Syme's prosthesis.

**Fig. 2:**
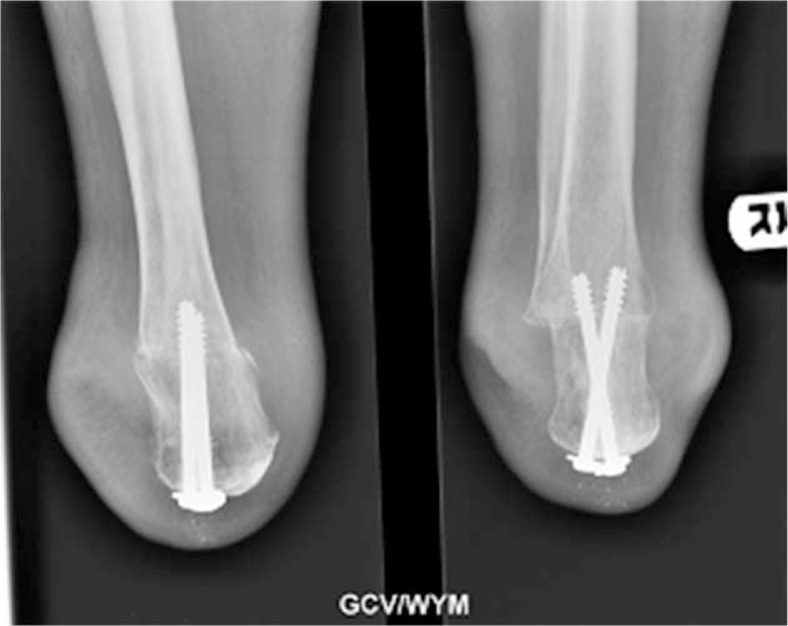
Radiographic views of a modified Pirogoff's stump at 6-month follow-up.

**Fig. 3:**
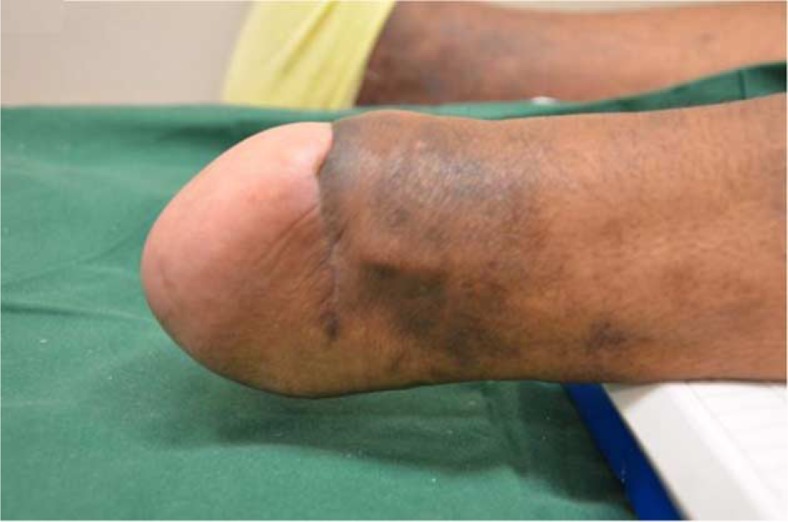
Clinical picture of a well-healed stump at 6-month follow-up.

Nather et al. (25) reported good outcomes in all six patients undergoing Pirogoff's amputation (Figs. 2 and 3) followed up over a minimum of 1 year. Strict selection criteria included a palpable posterior tibial pulse, ABI of more than 0.7, Hemoglobin level of more than 10 g/dL and serum albumin level of more than 30 g/L (25). The outcome of Pirogoff's amputation is still controversial. The cost of the prosthesis for Pirogoff's amputation is similar compared to that of a BKA. In terms of function, the Pirogoff's amputation is a weight-bearing stump. This has many advantages, including load sharing, which reduces the friction between the stump and the prosthesis and patients are able to ambulate short distances without wearing their prostheses. However, as the supramalleolar stump is bulbous in shape, it is difficult to fit a prosthesis for the Pirogoff's amputation.

## Discussion

Further sub-categorizing of operative methods is useful for being more accurate in the level of amputation for diabetic limb salvage surgery. Different levels of amputations provide unique problems in function and prostheses fitting due to the anatomical differences in each region. Problems in the midfoot will require muscle tendon transfers to ensure a well-functioning stump. Even though at times when a BKA stump is a better option for ambulation, patients may still choose a limb salvage operation in the attempt to preserve any remaining limb length.

## Conclusion

Minor amputations in patients with diabetic foot problems have been shown to be effective in limb salvage and reducing morbidity and mortality in patients. The authors have proposed several definitions regarding diabetic foot amputations while further studies are needed for a consensus on the definition on a successful versus non-successful diabetic limb salvage surgery.
